# Profiling of Non-Coding Regulators and Their Targets in Epicardial Fat from Patients with Coronary Artery Disease

**DOI:** 10.3390/ijms23105297

**Published:** 2022-05-10

**Authors:** Brendin Flinn, Christopher Adams, Nepal Chowdhury, Todd Gress, Nalini Santanam

**Affiliations:** 1 Department of Biomedical Sciences, Joan C Edwards School of Medicine, Marshall University, Huntington, WV 25755, USA; flinn9@marshall.edu; 2 Department of Cardiology, Joan C Edwards School of Medicine, Marshall University, Huntington, WV 25755, USA; adams111@marshall.edu; 3 Department of Cardiovascular and Thoracic Surgery, St. Mary’s Medical Center, Huntington, WV 25702, USA; nepal.chowdhury@st-marys.org; 4 Research Service, Hershel “Woody” Williams VA Medical Center, Huntington, WV 25704, USA; tgress@va.gov

**Keywords:** epicardial adipose tissue, non-coding RNAs, coronary artery disease, sex differences

## Abstract

Epicardial fat is a continuously growing target of investigation in cardiovascular diseases due to both its anatomical proximity to the heart and coronary circulation and its unique physiology among adipose depots. Previous reports have demonstrated that epicardial fat plays key roles in coronary artery disease, but the non-coding RNA and transcriptomic alterations of epicardial fat in coronary artery disease have not been investigated thoroughly. Micro- and lncRNA microarrays followed by GO-KEGG functional enrichment analysis demonstrated sex-dependent unique mi/lncRNAs altered in human epicardial fat in comparison to subcutaneous fat in both patients with and without coronary artery disease (IRB approved). Among the 14 differentially expressed microRNAs in epicardial fat between patients with and without coronary artery disease, the hsa-miR-320 family was the most highly represented. IPW lncRNA interacted with three of these differentially expressed miRNAs. Next-generation sequencing and pathway enrichment analysis identified six unique mRNAs–miRNA pairs. Pathway enrichment identified inflammation, adipogenesis, and cardiomyocyte apoptosis as the most represented functions altered by the mi/lncRNAs and atherosclerosis and myocardial infarction among the highest cardiovascular pathologies associated with them. Overall, the epicardial fat in patients with coronary artery disease has a unique mi/lncRNA profile which is sex-dependent and has potential implications for regulating cardiac function.

## 1. Introduction

Coronary artery disease (CAD) is a disorder of the coronary arteries characterized by the buildup of atherosclerotic plaques. Beyond obstruction of blood flow, these plaques can lead to other serious complications such as myocardial infarction and ischemic stroke [1]. CAD affects a large portion of the United States population, with 2017 rates showing an age-adjusted prevalence of 7.2% and 4.2% for males and females, respectively [2]. Investigations into the etiology, progression, and pathological mechanisms leading to CAD are thus needed to better understand both the causes of and potential treatments for CAD. A novel area of investigation that has a growing body of literature is how epicardial adipose tissue (EAT) is altered in patients with CAD and its role (if any) in the disease pathology.

EAT is a metabolically, physiologically, and anatomically distinct adipose tissue depot that is contiguous with the heart, being situated between the myocardium and the visceral layer of the serous pericardium (even extending into the myocardium without known negative clinical implications) [3]. EAT and the myocardium share the coronary microcirculation, allowing for paracrine interactions between the tissues [4]. Numerous potential EAT–cardiac interactions have been reported on, including pathological interactions (particularly EAT-mediated cardiac and vascular effects) in a variety of disease states, especially cardiometabolic disorders such as obesity, diabetes, and CAD [3,4,5,6,7,8,9,10,11,12]. The physiological significance of EAT in relation to the heart is yet to be fully elucidated, but two prominent roles are the potential for EAT to protect the heart from hypothermia (due to a possible increased thermogenic potential of EAT owing to brown adipocytes in the tissue) and the potential for EAT to supply fatty acids to the myocardium during times of high energy demand (since the myocardium primarily utilizes fatty acid metabolism, and since EAT is more lipolytic than other adipose tissue depots) [13,14,15].

A novel mechanism for EAT-mediated cardiac effects is the release of exosomes containing non-coding RNAs (ncRNAs), particularly microRNAs (miRNAs), that can subsequently be taken up by cardiomyocytes or vascular endothelial cells [4,16,17,18]. miRNAs are short, non-coding RNAs that have an average length of 22 nucleotides and generally act to repress the translation of target mRNA transcripts either by cleavage (when complementarity between the miRNA and its target sequence on the mRNA transcript is exact) or, more commonly, RNA interference and subsequent deadenylation leading to mRNA degradation; however, some ability to activate translation has also been demonstrated under certain conditions [19,20]. Though they are non-coding, miRNAs carry out important physiological functions via their interaction with other parts of the transcriptome, and hence altered regulation of miRNAs has been implicated in a variety of pathologies, with cancer being one of the most studied [21].

Characterization of the miRNA profiles of particular cell/tissue types during disease is useful both for elucidating novel pathological mechanisms and discovering novel biomarkers for disease states [22,23,24]. Due to EAT’s ability to impact cardiac function via the secretion of a variety of molecules that can be taken up by the heart and coronary circulation, characterization of the miRNA expression of EAT under pathological conditions can offer insights both into the differential transcriptomic regulation of EAT as well as how those same miRNAs could impact cardiac function. Thus, this characterization can elucidate both direct and indirect effects of EAT on cardiac function under pathological conditions stemming from altered miRNA expression.

Indeed, such characterization has been conducted in a variety of disease states already: hyperglycemia, atrial fibrillation, and CAD complicated by type 2 diabetes mellitus (T2DM) [25,26,27]. Interestingly, EAT volume itself has been shown to be correlated with differential expression of miRNAs even when controlling for confounding factors, making the use of miRNA expression as a biomarker for EAT volume a possibility [28]. Prior research has already demonstrated that the EAT of patients with CAD has a distinct gene expression profile [29]. Analysis of differences in the miRNA expression of the EAT of CAD vs. non-CAD patients can aid in giving a more detailed view of the altered transcriptomic regulation of EAT in patients with CAD. While some studies have begun to elucidate the altered miRNA profile of EAT in patients with CAD, further characterization and validation are necessary [30]. Transcriptomic regulation is further complicated by the presence of other ncRNAs that can influence gene expression at various levels of regulation. Long non-coding RNAs (lncRNAs) are a major example of these additional regulations. lncRNAs interact with both proteins and nucleic acids in a plethora of ways that alter gene expression: competitive interactions with histones that allow transcription of bound DNA, ‘guidance’ of proteins to either increase or decrease gene transcription, sequestration of RNA splicing proteins, and ‘sponging’ of miRNAs to allow for the translation of the mRNAs normally repressed by those miRNAs [31,32]. Of particular interest is its interactions with miRNAs since these interactions will alter gene expression and hence physiological pathways. Given the studies demonstrating altered miRNA regulation of EAT in pathological conditions, it is possible that lncRNAs could complicate the present understanding of such regulation.

Thus, the central goal of this investigation was to characterize both the miRNA and lncRNA profiles of EAT in the presence and absence of CAD. This will help determine the potential roles of both these ncRNAs in the altered transcriptomic regulation of EAT in patients with CAD.

## 2. Results

### 2.1. Differential Expression of miRNAs between EAT and SAT in CAD and Non-CAD Patients

**Figure 1:**
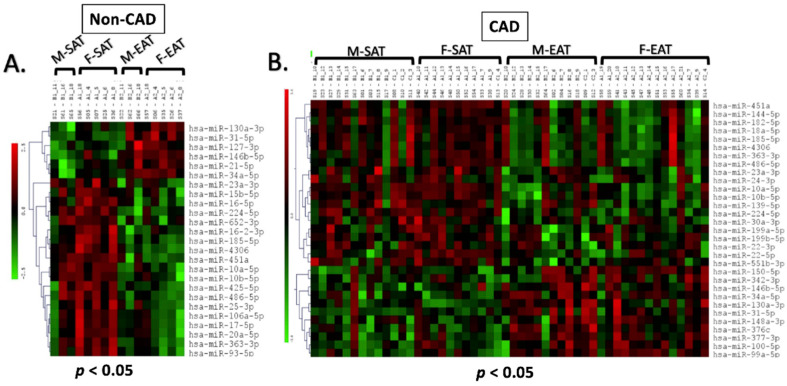
Heatmap of differentially expressed miRNAs between epicardial and subcutaneous adipose tissue samples from patients with and without CAD: microRNA microarray on EAT and SAT samples from both males and females were analyzed and compared. The heatmap (LC Sciences, Houston, TX, USA) displayed shows differentially expressed microRNAs between EAT and SAT in non-CAD patients (**A**) and CAD patients (**B**). Differential analysis was first performed using ANOVA to produce a miRNA expression profile overview across all samples. This was followed by *t*-tests *p* < 0.05, to identify significantly differentiated miRNAs between EAT and SAT in patients with and without CAD.

miRNA microarray revealed differential expression of miRNAs between EAT and subcutaneous adipose tissue (SAT) in both CAD and non-CAD patients. Comparing the EAT (n = 3 males and n = 5 females) and SAT (n = 3 males and n = 5 females) in the non-CAD patients revealed 25 differentially expressed miRNAs (DEmiRNAs; *p* < 0.05) (Figure 1A) of which 13 miRNAs were downregulated, 3 miRNAs were upregulated, and 8 miRNAs, though significant, had low abundance. In contrast, 31 miRNAs were significantly (*p* < 0.05) differentially expressed between EAT (n = 12 males and n = 14 females) and SAT (n = 13 males and n = 11 females) in patients with CAD (Figure 1B). Fourteen miRNAs were downregulated, 8 miRNAs were upregulated, and 9 miRNAs were significant but had low abundance (Table S1).

**Figure 2:**
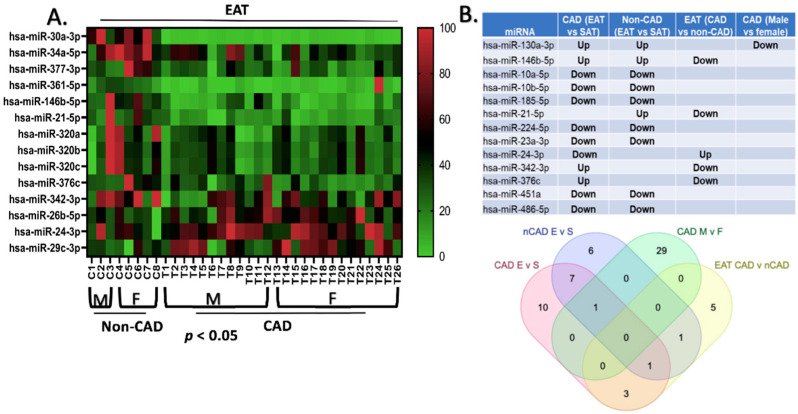
Heatmap of differentially expressed miRNAs in EAT between CAD and non-CAD: microRNA microarray on EAT from patients with and without CAD (both males and females) were compared. Fourteen microRNAs were significantly altered (expression data points for each miRNA were normalized on a 0–100 scale with 0 (green), being the lowest expression and 100 (red) being the greatest). hsa-miR-29c-3p, hsa-miR-24-3p, and hsa-26b-5p were upregulated in the EAT of patients with CAD (**A**). List of microRNAs that were similarly altered in EAT vs. SAT in patients with or without CAD and their sex specificity is displayed in the table and lotus matrix (**B**). Only miRNAs with expressions that were found to be significantly different (*p* < 0.05) in unpaired *t*-test are presented. The following groups of comparisons were included: CAD (EAT vs. SAT) = epicardial adipose tissue (EAT or E) versus subcutaneous adipose tissue (SAT or S) in patients with CAD (‘up’ indicates greater and ‘down’ indicates lower expression in EAT); nCAD (EAT vs. SAT) = epicardial adipose tissue versus subcutaneous adipose tissue in patients without CAD (‘up’ indicates greater and ‘down’ indicates lower expression in EAT); EAT (CAD vs. nCAD) = epicardial adipose tissue from patients with CAD versus epicardial adipose tissue from patients without CAD (‘up’ indicates greater and ‘down’ indicates lower expression in EAT of patients with CAD); CAD (male vs. female) = males versus females in patients with CAD (‘up’ indicates greater and ‘down’ indicates lower expression in females).

Comparing the EAT of CAD patients (n = 12 males and 13 females) to the EAT of non-CAD patients (n = 3 males and 5 females) revealed 14 DEmiRNAs that were statistically significant (*p* < 0.05) (Figure 2A). The miR-320 family was the most highly represented, with hsa-miR-320a, hsa-miR-320b, and hsa-miR-320c all being downregulated in the EAT of CAD patients. Two miRNAs, hsa-miR-146b-5p and hsa-miR-21-5p, reached a significance of *p* < 0.01. Four miRNAs had low average abundance (hsa-miR-30a-3p, hsa-miR-34a-5p, hsa-miR-377-3p, and hsa-miR-361-5p). Among the DEmiRNAs, there were 9 miRNAs that were similarly differentially expressed and regulated in EAT vs. SAT between the non-CAD and CAD patients, hsa-miR-130a-3p; hsa-miR-146b-5p were upregulated whereas hsa-miR-10a-5p; hsa-miR-10b-5p; hsa-miR-185-5p; hsa-miR-224-5p; hsa-miR-23a-3p; hsa-miR-451a; hsa-miR-486-5p were all downregulated (Figure 2B). hsa-miR-21-5p was upregulated in EAT vs. SAT in non-CAD patients but downregulated in EAT when compared between CAD and non-CAD patients. Similarly, hsa-miR-24-3p was downregulated and hsa-miR-342-3p and hsa-miR-376C were upregulated in EAT vs. SAT in CAD patients, whereas the former miRNA was upregulated and the latter two miRNAs were downregulated in EAT when compared between CAD and non-CAD patients (Figure 2B).

**Figure 3:**
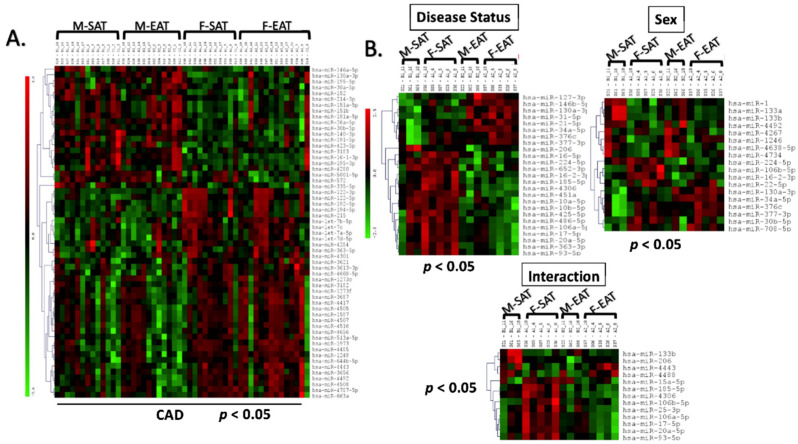
Sex specific differences in miRNA regulation in EAT and SAT from patients with CAD: microRNA microarray on EAT and SAT samples from patients with CAD showed sex-specific differences in expression. Unpaired *t*-test (*p* < 0.05) significantly altered microRNAs are displayed (**A**). Two-way ANOVA showed significant differences in microRNAs and interactions between both sex and disease status and interactions were observed (**B**).

Comparing miRNA expression between males and females showed 57 DEmiRNAs (*p* < 0.05) in the CAD condition (Figure 3A). Two-way ANOVA (using sex and disease status) showed significant differences in miRNA expression and an interaction between disease status and gender (Figure 3B).

### 2.2. Functional Pathway Analysis Reveals Potential Roles for the DEmiRNAs in the EAT of CAD Patients

**Figure 4:**
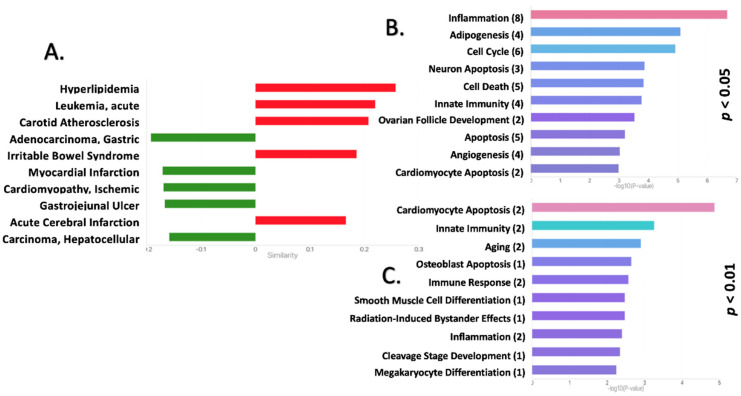
Functional pathways and disease correlations targeted by the DEmiRNAs in EAT: TAM 2.0 identified both disease pathologies (**A**) and functional pathways (**B**), associated with the miRNAs differentially expressed (all with *p* < 0.05) in EAT from patients with and without CAD as well as the functional pathways associated with the two miRNAs that had *p* < 0.01 (**C**); (functional association strength is expressed as the-log_10_ (*p*) along the *x*-axis). Negative similarity scores in panel **A** (green) indicate that the regulation of the miRNAs was opposite to the normal regulation seen in the disease pathology while positive scores (red) indicated that the regulation of the miRNAs was the same direction as the regulation normally seen in the disease pathology. Coloration of the bars in panels (**B**,**C**) is for stylization and not meant to reflect particular values. Cardiac diseases and cardiomyocyte apoptosis had the highest correlations.

Using the publicly available bioinformatics tool TAM 2.0 [33] (which uses published literature on miRNA-associated functions and pathologies rather than target prediction) to correlate the DEmiRNAs with both functional pathways and disease pathologies demonstrated various potential roles for miRNAs in the EAT of CAD patients (Figure 4). Metabolic- and cardiovascular-related conditions such as ischemic cardiomyopathy, acute cerebral infarction, myocardial infarction, and carotid atherosclerosis were among the top hits for disease pathologies associated with the DEmiRNAs (Figure 4A). Overall, the correlations with these pathologies indicate that the deregulation of miRNAs in the EAT of CAD patients could play a similar role as the deregulation of these miRNAs in other metabolic- and cardiovascular-related conditions, implicating the miRNA profile of EAT as a potential mechanism of either CAD progression or protection from deleterious alterations in CAD. Further, the determination of associations between the DEmiRNAs of the EAT of CAD patients and functional pathways using TAM 2.0 revealed particular physiological roles that the deregulated miRNA profile of CAD patients EAT could have. Inflammation, adipogenesis, and cell cycle regulation were the most associated functional pathways for the DEmiRNAs (Figure 4B). Performing the same analysis using only the two miRNAs with significance levels of *p* < 0.01 (hsa-miR-146b-5p and hsa-miR-21-5p) produced cardiomyocyte apoptosis as the most associated functional pathway (Figure 4C).

### 2.3. GO and KEGG Functional Enrichment Analyses of the DEmiRNAs in EAT

**Figure 5:**
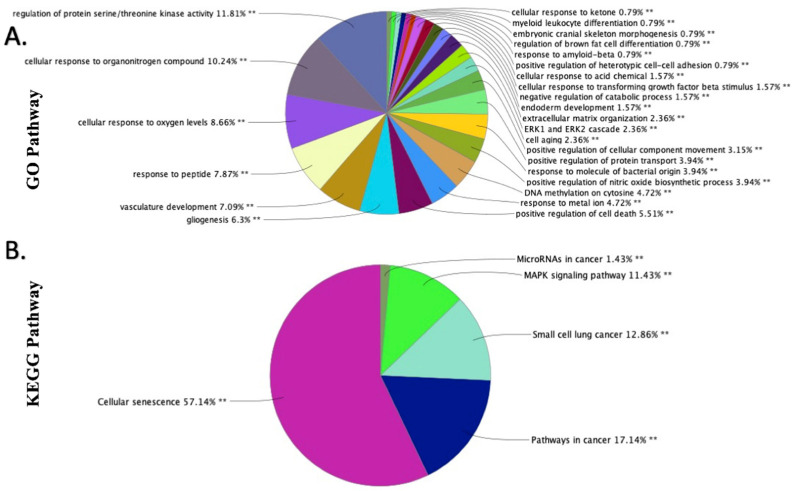
GO and KEGG functional enrichment analysis of DEmiRNAs: GO enrichment analysis shows the biological processes and cellular components of the DEmiRNAs in the EAT of CAD patients (**A**). KEGG enrichment analysis shows the molecular functions associated with the DEmiRNAs (**B**). ClueGO v2.5.8 and CluePedia v1.5.8 in Cytoscape v3.9.0 were used for enrichment analysis. ** indicates *p* < 0.01 for the associations.

For more specific functional enrichment associations, correlation of the DEmiRNAs to GO categories and KEGG pathways was performed using ClueGo v2.5.8 and CluePedia v1.5.8 in Cytoscape v3.9.0 [34,35]. The determination of mRNA targets of the DEmiRNAs in EAT was performed using the miRTarBase dataset, a curation of experimentally verified miRNA–mRNA interactions [36]. CluePedia and ClueGo (using the miRTarBase dataset) produced a broad network of mRNA targets, many of which were only targeted by single miRNAs, but a substantial portion of which were targeted by multiple of the DEmiRNAs (Figure S1). Correlation with GO categories showed enrichment of the following biological processes or cellular components associated with the mRNAs targeted by the DEmiRNAs: regulation of protein serine/threonine kinase activity, cellular response to organonitrogen compound, cellular response to oxygen levels, response to peptide, and vasculature development (Figure 5A and Figures S2 and S3). Correlation with KEGG pathways showed enrichment of the functional pathways associated with the mRNAs targeted by the DEmiRNAs: cellular senescence, pathways in cancer, small cell lung cancer, MAPK signaling pathway, and microRNAs in cancer (Figure 5B and Figures S4 and S5).

### 2.4. Pathway Enrichment Analysis of DEmiRNAs and DEmRNA Interactions in EAT

**Figure 6:**
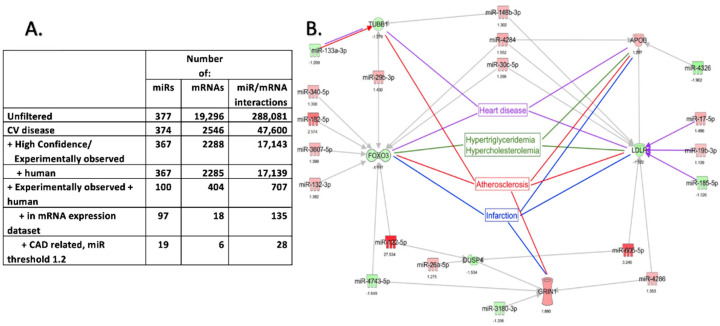
Pathway enrichment analysis of the DEmiRNAs and DEmRNA interactions from EAT and SAT: Ingenuity Pathway Analysis (IPA) was used to perform pathway enrichment analysis on the mRNA (RNA-seq) and miRNA (microRNA microarray) that were differentially expressed in EAT compared to SAT. Targets of miRNAs relevant to cardiovascular diseases were identified after a stringent filtering process (**A**). mRNA targets of DEmiRNAs are shown with IPA annotations for selected cardiovascular disorders (**B**). Six miRNA–mRNA pairs that played a role in cardiovascular disease were identified.

Next-generation sequencing (NGS) was performed, and data analysis revealed 125 differentially expressed mRNAs (DEmRNAs) (*p* < 0.05) between the EAT and SAT of the patients sampled. The targets of the DEmiRNAs were then detected in the DEmRNAs dataset. Pathway enrichment analysis was performed on the RNA sequencing data and miRNA microarray data using Ingenuity Pathway Analysis (IPA). The targets of miRNAs relevant to cardiovascular diseases (CVDs) were further identified using TargetScan after a stringent filtering process (Figure 6A) [37]. mRNA targets of differentially expressed miRNAs with IPA annotations for selected cardiovascular disorders are shown in (Figure 6B). The colored arrows for miRNAs denote their cardiovascular annotations. Six miRNA–mRNA pairs (some of the mRNAs were targeted by multiple miRNAs) that played a role in CVD were identified. The potential interactions between the two datasets found that the EAT transcriptome is involved in cardiovascular-related pathologies (atherosclerosis, hypertriglyceridemia, hypercholesterolemia, and myocardial infarction) (Figure 6B) and identified genes such as DUSP4, TUBB1, LDLR, FOXO3, APOB, and GRIN1 as major targets of possible interest.

### 2.5. qPCR Validation of the Differentially Expressed mRNAs in the EAT of Patients with CAD

qPCR of RNA isolated from the EAT of patients with and without CAD (validation cohort) demonstrated that the mRNAs DUSP4 and FOXO3 had statistically significant (*p* < 0.0001) upregulation in the EAT of patients with CAD (Figure S6A). Both mRNAs had a >200-fold upregulation, demonstrating significant CAD-induced differential expression of the two mRNAs in EAT. This confirms the distinct involvement of these mRNAs in the pathophysiology of EAT (in comparison to SAT) found in the NGS results.

### 2.6. qPCR Validation of the Differentially Expressed miRNAs in the EAT of Patients with CAD

qPCR of miRNAs isolated from the EAT of patients with and without CAD (validation cohort) demonstrated that the miRNAs hsa-21-5p and hsa-320a had statistically significant (*p* < 0.03 and *p* < 0.01, respectively) downregulation in the EAT of patients with CAD (Figure S6B). This confirms the downregulation of these miRNAs found in the miRNA array results. hsa-146b-5p and hsa-26b-5p both also showed downregulation in the EAT of patients with CAD in the qPCR, but the changes were not statistically significant (Figure S6B).

### 2.7. LncRNA Array Shows Differential Expression of Long Non-Coding RNAs

**Figure 7:**
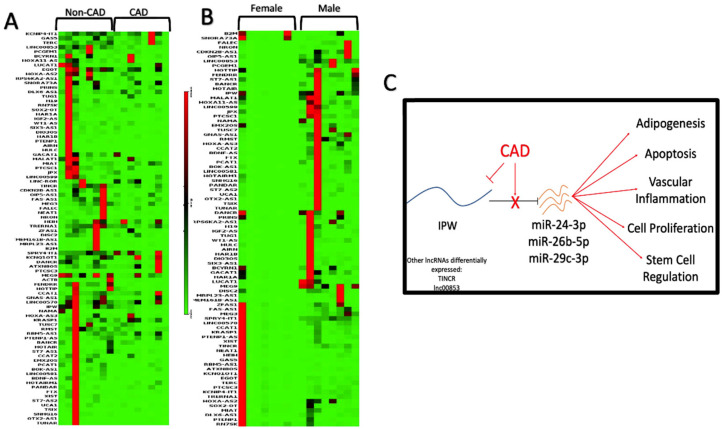
DElncRNAs in EAT: LncRNA array and data analysis was conducted using Qiagen GeneGlobe analysis tool. Red: higher expression; green: low expression. DElncRNAs expression for male patients with or without CAD. The lncRNAs with statistically significant differential expression were TINCR, IPW, and LINC00853 (**A**). Sex differences in DElncRNAs in patients without CAD. The lncRNAs with statistically significant differential expression were RMST, MEG9, and LINC00853 (**B**). lncRNA–miRNA interactions were determined in the DElncRNAs in EAT based on CAD status in males. In CAD male patients, IPW downregulation leads to an increase in the levels of the miRNAs hsa-miR-24-3p, hsa-miR-26b-5p, and hsa-miR-29c-3p, associated with adipogenesis, apoptosis, vascular inflammation, cell proliferation, and stem cell regulation as determined by TAM 2.0 (**C**).

The lncRNA PCR arrays showed three lncRNAs were significantly downregulated in the EAT of male patients with CAD compared to EAT from patients without CAD: IPW, TINCR, and linc00853. DIANA-LncBase v3.0 did not show any interactions with the DEmiRNAs for TINCR or linc00853 but showed that three of the DEmiRNAs had interactions with IPW: hsa-miR-24-3p, hsa-miR-26b-5p, and hsa-29c-3p. As observed by the miRNA microarray analysis, all three of these miRNAs were upregulated in the EAT of patients with CAD. Thus, the data suggest that the downregulation of IPW in the EAT of male patients with CAD may lead to a subsequent upregulation in the three miRNAs that showed interactions with IPW (Figure 7). Functional analysis using TAM 2.0 showed significant functional associations for the three miRNAs interacting with IPW: adipogenesis, apoptosis, vascular inflammation, cell proliferation, and stem cell regulation (Figure 7).

In the EAT of female patients, lncRNA arrays did not show any differential lncRNA expression with statistical significance between CAD and non-CAD patients. There were also no differentially expressed lncRNAs between the EAT of male and female CAD patients. However, there did exist three DElncRNAs when comparing the EAT of male and female non-CAD patients, all of which were upregulated in males in comparison to females: RMST, MEG9, and linc00853. DIANA-LncBase v3.0 did not show any experimentally verified human miRNA interactions for RMST or MEG9 but did show that linc00853 interacts with hsa-miR-1180-3p, though it was not detected in the DEmiRNAs in EAT of male and female patients without CAD.

## 3. Discussion

Increased prevalence of obesity rates in the United States has increased the burden of heart diseases including CAD. Recent studies have suggested that increased EAT volumes are associated with the severity of CAD [38,39]. Hence, determining functional changes in EAT compared to other adipose depots in patients with CAD compared to those without will provide a better understanding of the role of this fat in heart disease and identify novel targets for diagnostics or treatment for CAD. In this study, we assessed ncRNA (miRNA and lncRNA) changes in EAT versus SAT obtained from both patients with and without CAD. We also assessed if there were sex differences in the differentially expressed ncRNAs. Finally, we correlated the DEmiRNAs to the DEseq data and DElncRNA to DEmiRNAs in these tissues to identify any interactions that might be of significance in CAD. We also used pathway analysis tools for target prediction and physiological/pathological pathways that the DEmiRNAs could potentially target.

Using RNA sequencing as well as microRNA microarray analysis showed that both the mRNA and miRNA expression profiles differed between EAT and SAT. These findings were consistent with the long line of literature pointing towards a distinct EAT phenotype [3,10,15,40,41]. Importantly, IPA analysis of the DEmRNA and DEmiRNAs of the EAT of CAD patients demonstrated the potential involvement of miRNAs with cardiovascular pathologies. The appearance of atherosclerosis in the hit targets as a pathology related to the DEmRNA–DEmiRNA interactions was particularly relevant because it points towards altered regulation of EAT transcriptome in CAD. Also of note was the appearance of hypertriglyceridemia and hypercholesteremia, two lipid dysregulations shown to be associated with diseased EAT, further confirming the unique relation of EAT to cardiovascular and metabolic pathways in comparison to SAT [42,43]. Additionally, gene targets such as FOXO3, LDLR, and APOB are well studied in CVDs [44,45]. Adiponectin promotes macrophage autophagy by suppressing Akt/FOXO3 signaling [46]. Tubulin beta 1 (TUBB1) is highly expressed in platelet and megakaryocyte microtubules [47]. Polymorphism in TUBB1 (Q43P) has been shown to protect men from CVD [48]. Dual Specificity Phosphatase 4 (DUSP4) modulates Akt and p38 kinase pathways. DUSP4 is considered a protective target in cardiomyopathy and other CVDs [49,50]. By modulating p38 kinase activity, DUSP4 is considered a good target for the treatment of myocardial infarction [51]. GRIN1 is a major subunit of the N-methyl-D-aspartate receptors (member of the glutamate receptor channel superfamily). It regulates the excitatory firing rate of cardiomyocytes [52]. Dextromethorphan, a GRIN1 antagonist, has been shown to inhibit neo-intima and atherosclerosis formation in mice [53,54] and has shown promise as an adjunct treatment for diabetes [55]. Interestingly, GRIN1 and DUSP4 directly interact with each other and can modulate each other’s expression [56]. Overall, the data presented here further confirm that EAT is distinct from SAT in ways that implicate it in cardiovascular conditions, including CAD.

The global functional enrichment pathways targeted by the miRNAs that were differentially expressed in the EAT of CAD patients compared to non-CAD patients, determined by various bioinformatics tools such as TAM 2.0, KEGG, and GO, included changes in metabolism, immune responses, inflammation, and cardiac function (such as association with cardiovascular pathologies and cardiomyocyte apoptosis). All of these findings are in line with the growing conception of EAT as a metabolic center with both paracrine and endocrine functions [57,58]. The appearance of atherosclerosis as a pathology associated with the miRNAs differentially expressed in the EAT of CAD patients was expected given that CAD normally results from the buildup of atherosclerotic plaques within the coronary arteries. The appearance of myocardial infarction as highly associated with the DEmiRNAs, however, points towards a rather novel relationship between EAT and cardiac function, especially as it relates to CAD. It could be the case that EAT in patients with CAD could release an altered miRNA profile into the local circulation, including miRNAs that induce direct cardiac effects, such as ones that are associated with myocardial infarction or cardiomyocyte apoptosis (hsa-miR-320 family, miR-146b-5p, and hsa-miR-499) [59,60,61,62]. Changes in miRNAs associated with immune system and inflammatory mediators (such as hsa-miR-320 family and hsa-miR-21) potentially point towards a role for miRNA regulation in EAT-mediated CAD progression (since those mediators play integral roles in CAD progression, and EAT has been implicated in such progression) [7,63,64,65]. The appearance of lipid regulation pathways as associated with the DEmiRNAs is interesting as EAT has been considered a producer of free fatty acids (due to its being highly lipolytic) for the heart; thus, CAD may affect the metabolic relationship between EAT and the heart via miRNA regulation [14]. hsa-24-3p, hsa-miR-378, and hsa-miR-33, for instance, have been shown to regulate lipolysis in adipose tissue, suggesting that changes in their expression in EAT could alter the supply of free fatty acids in the local circulation for the heart [66,67,68]. Previous reports identified elevated hsa-miR-34a as a potential biomarker for CAD with high clinical significance both in EAT and plasma [69,70]. However, our samples showed a statistically significant downregulation of hsa-miR-34a-5p (*p* < 0.05). This downregulation may be attributable to a few factors. First, all samples gave very low abundance levels for hsa-miR-34a-5p. Secondly, the patient population utilized for this investigation may have differed from those used in the previous investigations in ways that altered hsa-miR-34a-5p expression.

In contrast to previous reports, we found a downregulation of the hsa-miR-320 family members (a/b/c) in the EAT of CAD patients. In adipocytes, hsa-miR-320 has been shown to suppress insulin signaling through the PI3K pathway, potentially leading to increased lipolysis, decreased glucose uptake, and reduced lipogenesis [71,72]. hsa-miR-320 family members have also been shown to be positively correlated with endoplasmic reticulum stress and inflammatory mediator production in adipocytes [73]. In terms of cardiac function, miR-320 family members have been shown to reduce cardiac survival, induce cardiomyocyte apoptosis, and lead to lipotoxicity-induced cardiac dysfunction stemming from increased fatty acid uptake in diabetic mice and humans [59,74,75]. Interestingly, the latter effect of miR-320 family members was shown to result from transcriptional induction of the *CD36* gene (fatty acid translocase) [74]. Importantly, the ability of miRNAs to affect different cell types in different ways is implicated in the interaction between miR-320 family members and cardiac function. Recently, cardiac miR-320 family members have been shown to be deleterious in cardiomyocytes but protective in cardiac fibroblasts [61]. The plethora of observed functions associated with the miR-320 family provides ample ways in which the downregulation of its members in EAT could affect both EAT function and cardiac function (indirectly and directly). For EAT itself, miR-320 family downregulation in CAD may increase insulin sensitivity, leading to reduced lipolysis, increased glucose uptake, and increased lipogenesis; however, the study demonstrating miR-320 family effects in adipocytes was conducted on white adipocytes. However, EAT is considered to be “beige-like” due to there being brown adipocytes heterogeneously embedded within the tissue, introducing the possibility that miRNA regulation in EAT could differ from other adipose depots [15,40]. The possible decreased lipolysis could indirectly affect cardiac function since there would be less fatty acid release from EAT, reducing local supplies for the myocardium [14]. There is also the possibility that miR-320 family members (along with other miRNAs) could be released into the coronary microcirculation by EAT, being transferred to the myocardium; this leaves the question of how a reduced uptake of miR-320 family members by cardiac cells resulting from a downregulation of miR-320 family members in EAT would affect cardiac function. The dual effects of miR-320 in cardiac function (having different effects in different cardiac cells) complicate this question since miR-320 could have both positive and negative effects on the cardiac system under stress from CAD. For instance, reduced miR-320 family members in cardiomyocytes could reduce cardiomyocyte apoptosis, but reduced miR-320 in cardiac fibroblasts could promote hypertrophy [61]. Overall, the possible roles of miR-320 family members and other DEmiRNAs identified in both the EAT and EAT–cardiac interactions of patients with CAD are extensive and require mechanistic studies in the future.

The observation of differential miRNA expression between the EAT of males and females only in patients with CAD but not without CAD suggests possible sex-dependent changes in miRNA expression resulting from CAD or other sex-dependent factors. Sexual dimorphisms in miRNA expression have been documented in a variety of pathologies, including cardiac pathologies [76,77]. We have previously reported on sex-dependent changes in both mRNA and miRNA expression in EAT (related to obesity and aging, respectively), lending credence to the possibility of sex-dependent changes in EAT miRNA expression in CAD [78,79]. The significance resulting from two-way ANOVA (using sex and disease status) suggests an interaction between CAD status and sex in EAT miRNA expression. Future studies need to better elucidate the influence of sex on the miRNA expression in the EAT of patients with CAD as well as other cardiovascular conditions.

The differential expression of multiple lncRNAs in the EAT of male CAD patients adds another layer to the possible transcriptomic alterations occurring under pathological conditions. The limitation of this study was the use of an RT^2^ PCR array, which only detects 84 lncRNAs, and not sequencing technologies. The finding that downregulation of IPW led to upregulation of the miRNAs (microRNA microarray data) known to interact with it (hsa-miR-24-3p, hsa-miR-26b-5p, and hsa-miR-29c-3p) supports the idea that lncRNAs in EAT may be involved in regulating miRNAs. However, validation of the target miRNAs did not support the directionality of the lncRNA regulation, which can be attributed to the variability in the patient samples used and the sample sizes tested. The functions associated with the DEmiRNAs that interact with IPW point towards the potential roles of EAT in either mediating or protecting against CAD-induced pathological alterations (Figure 7). Vascular inflammation particularly stands out as it is a component of CAD progression [1]; meaning, the miRNAs in EAT could be involved in one of the critical components of CAD progression. Importantly, EAT has been shown to be involved in mediating inflammation [80], and thus, the data here suggest lncRNAs and miRNAs as possible factors influencing the alterations in EAT that aid in CAD progression.

Our study further validates the unique nature of EAT that implicates it in cardiovascular function, particularly under pathological conditions. We show that EAT in CAD patients has an altered non-coding RNA (miRNA and lncRNA) profile that points towards altered functional regulation in this tissue during CAD. The differential expression of the non-coding RNAs can also be influenced by the clinical characteristics of the patient cohorts which needs to be further explored. Future studies should evaluate the changes in DEmi/lncRNAs–DEmRNA interactions to obtain a larger picture of the functional alterations in EAT in CAD. Larger samples may also be useful for evaluating any potential sex-specific changes in these alterations. Overall, we find that the altered non-coding RNA regulation in EAT may be a part of the pathophysiology of CAD.

## 4. Materials and Methods

### 4.1. Sample Collection and Clinical Characteristics

Samples of epicardial adipose tissue (EAT) and subcutaneous adipose tissue (SAT) were obtained from a total of 61 patients (36 males and 24 females) who either had CAD (n = 42) and were undergoing coronary artery bypass graft (CABG, 26 males and 16 females) or did not have CAD (n = 18) and were undergoing aortic valve replacement/repair (AVR, 11 males and 7 females). All samples were obtained from St. Mary’s Medical Center, Huntington, WV, USA, and were frozen immediately. The collection and use of these samples for this study were approved by the Marshall University Human Investigations Committee (IRB). All patients involved were consented, and all applicable HIPAA regulations were followed. The average age was, males = 65 ± 2 years (range: 28–80 years) and females = 58 ± 2 years (range: 43–73 years). The average BMI was, males = 30.8 ± 1.2 kg/m^2^ (range: 21.79-63.26 kg/m^2^) and females = 33.1 ± 1.3 kg/m^2^ (range: 19.9–46.3 kg/m^2^). The baseline average LDL for males = 96.9 ± 6.9 mg/dL (range: 51–218 mg/dL) and females = 101.1 ± 6.4 mg/dL (range: 44–154 mg/dL) and average triglyceride levels for males = 165.2 ± 16.6 mg/dL (range: 52–436 mg/dL) and for females = 172.2 ± 18.6 mg/dL (range: 62–382 mg/dL).

### 4.2. miRNA Microarray of EAT and SAT

miRNAs were isolated from both the SAT and EAT of all patients using a commercially available miRNA isolation kit (QIAGEN, Germantown, MD, USA). A miRNA microarray was performed (LC Sciences, Houston, TX, USA) on EAT (n = 12 males with CAD and n = 3 non-CAD; n = 14 females with CAD and n = 5 non-CAD) and SAT (n = 13 males with CAD and n = 3 non-CAD; n = 11 females with CAD and n = 5 non-CAD) samples to determine differentially expressed miRNAs (DEmiRNAs). The microRNA microarray data analysis was performed by LC Sciences which includes image digitization, background subtraction, signal significance analysis, normalization, and differential analysis. The image digitization was performed using the “Array-Pro Analyzer” (MediaCybernatics). After background subtraction and signal significance analysis, normalization was performed using LOWESS (locally weighted scatterplot smoothing) method. Since we had several groups of samples, the differential analysis was first performed using ANOVA to produce a miRNA expression profile overview across all samples. This was followed by *t*-tests to identify significantly differentiated miRNA combinations of two groups of interest. Unpaired *t*-test was used to determine the DEmiRNAs between the EAT and SAT using the data collected from all patients. Unpaired *t*-test was also used to determine the DEmiRNAs in both the EAT and SAT between patients with and without CAD and between males and females. TAM 2.0 was used to correlate the DEmiRNAs, comparing the EAT of CAD patients to non-CAD patients, with holistic functional pathways and pathologies [33]. ClueGO v2.5.8 and CluePedia v1.5.8 were used in Cytoscape v3.9.0 to find the mRNA targets of the DEmiRNAs and to determine the Gene Ontology (GO) and KEGG pathway associations for those targets [34,35,81].

### 4.3. Next-Generation Sequencing of EAT and SAT

RNA isolated from the EAT and SAT of CAD (n = 10) and non-CAD (n = 4) was subjected to NGS using an Illumina 1500 (Illumina, San Diego, CA, USA). Reads were aligned using TopHat and counted using Rsamtools. DEseq was used to determine the differential expression of mRNAs. Ingenuity Pathway Analysis (IPA) (Qiagen, Germantown, MD, USA) was used to correlate the differentially expressed mRNAs (DEmRNAs) with miRNAs and pathologies.

### 4.4. LncRNA Array

EAT samples from 16 males (n = 8 CAD and n = 8 non-CAD) and 16 females (n = 8 CAD and n = 8 non-CAD) were used to determine the expression of lncRNAs in patients with and without CAD. Total RNA was isolated from the samples using QIAzol lysis reagent (QIAGEN, Germantown, MD, USA) to lyse the adipocytes followed by isolation of the RNA using an miRNeasy kit (QIAGEN, Germantown, MD, USA). Complementary DNA (cDNA) was prepared from the isolated RNA via reverse transcription. The prepared cDNA was plated into 384-well plates (4 samples per plate) containing primers for 84 different long non-coding RNAs (LAHS-001Z, RT^2^ lncRNA PCR Array Human lncFinder, QIAGEN, Germantown, MD, USA) and qPCR was conducted using a Roche LightCycler 480 II (Roche Sequencing and Life Sciences, Wilmington, MA, USA) to determine the differential expression of the lncRNAs. Statistical analysis for differential expressed lncRNAs (DElncRNAs) was conducted using the Qiagen Geneglobe analysis tool specific for the lncRNA array used. DIANA-LncBase v3.0 was used to determine the miRNAs potentially ‘sponged’ by the DElncRNAs, i.e., DEmiRNA–DElncRNA interactions [82]. The targets of the DElncRNAs were compared to the DEmiRNAs to determine potential lncRNA-mediated changes in miRNA expression in the EAT of CAD patients. For matches between the two datasets, the direction of regulation of the DElncRNAs was compared to the direction of regulation of the DEmiRNAs; the pairings with opposite directionality (e.g., upregulation of the DElncRNA and downregulation of its miRNA target) were considered potential lncRNA–miRNA interactions that could affect the translation of mRNAs targeted by miRNAs. The DEmiRNAs selected from this process were analyzed using TAM 2.0 to determine cellular functions that may be subject to regulation by lncRNA-miRNA interactions in EAT.

### 4.5. qPCR Validation of DEmRNAs

qPCR was conducted on 12 new samples of EAT (n = 6 CAD, and n = 6 non-CAD) to validate select target mRNAs (DUSP4 and FOXO3) found through NGS. Total RNA was isolated from the EAT of all patients using a commercially available miRNA isolation kit (QIAGEN, Germantown, MD, USA). Reverse transcription of the total RNA was conducted using the commercially available iScript cDNA Synthesis Kit (Bio-Rad, Hercules, CA, USA). Forward and reverse PCR primers for DUSP4 (TAGTACAGGCCTCTAGCCCCA and TGCAATATTGACATCCCCCGA, respectively) and FOXO3 (GAGGCCGTCGATTCGCTC and CAGGAGGACCTGAAGACGT, respectively) were obtained from Invitrogen (Waltham, MA, USA). qPCR was conducted on the resultant cDNA on an Applied Biosystems StepOnePlus Real-Time PCR System (Waltham, MA, USA).

### 4.6. qPCR Validation of DEmiRNAs

qPCR was conducted using 12 new samples of EAT (n = 6 CAD and n = 6 non-CAD) to validate select DEmiRNAs (hsa-146b-5p, hsa-26b-5p, hsa-21-5p, hsa-320a) in the EAT of patients with CAD. miRNAs were isolated from the EAT of all patients using a commercially available miRNA isolation kit (QIAGEN, Germantown, MD, USA). Reverse transcription of the miRNAs was conducted using the miRCURY LNA RT Kit (QIAGEN, Germantown, MD, USA). PCR primers for hsa-miR-146b-5p, hsa-miR-26b-5P, hsa-miR-21-5p, and hsa-miR-320a were obtained from Qiagen. qPCR was conducted on a CFX Connect Real-Time System (Bio-Rad, Hercules, CA, USA) using the cycle settings recommended in the miRCURY LNA RT Kit (QIAGEN, Germantown, MD, USA). ΔC*_t_* values were determined for each miRNA in each sample using the spliceosomal RNA U6 for normalization. Unpaired *t*-test (using ΔC*_t_* values) was used to determine statistically significant differential expression of the miRNAs between patients with and without CAD.

## Supplementary Materials

The following are available online at https://www.mdpi.com/article/10.3390/ijms23105297/s1.
