# Supplementary material for: Profiling of Non-Coding Regulators and Their Targets in Epicardial Fat from Patients with Coronary Artery Disease
Source: Int J Mol Sci. 2022 May 10;23(10):5297. doi: 10.3390/ijms23105297 (PMC9141930; doi:10.3390/ijms23105297)
Supplement: Supplementary file 1 [file ijms-23-05297-s001.zip › ijms-1692202-supplementary.pdf]

TABLE S1: *List of significantly altered microRNAs in the following groups*: (i) EAT versus SAT in CAD patients (both males and females); (ii) EAT versus SAT in Non-CAD patients (both males and females; (iii) EAT in CAD versus non-CAD patients and (iv) EAT/SAT in male versus female CAD patients.

CAD EAT vs. SAT	*p* Value	Non-CAD EAT vs. SAT	*p* Value	EAT CAD vs. non-CAD	*p* Value	CAD M vs. F	*p* Value
hsa-miR-10b-5p	3.46E-05	hsa-miR-4306	3.46E-04	hsa-miR-146b-5p	2.58E-05	hsa-miR-122-5p	1.72E-06
hsa-miR-224-5p	3.49E-05	hsa-miR-224-5p	1.65E-03	hsa-miR-21-5p	1.37E-04	hsa-miR-16-1-3p	8.43E-06
hsa-miR-10a-5p	3.72E-05	hsa-miR-486-5p	2.16E-03	hsa-miR-34a-5p	1.35E-03	hsa-miR-140-3p	2.44E-05
hsa-miR-150-5p	7.21E-05	hsa-miR-10a-5p	2.28E-03	hsa-377-3p	1.52E-02	hsa-miR-152	3.64E-05
hsa-miR-31-5p	1.88E-04	hsa-miR-185-5p	2.46E-03	hsa-miR-320a	1.77E-02	hsa-miR-192-5p	6.08E-05
hsa-miR-130a-3p	3.97E-04	hsa-miR-363-3p	2.48E-03	hsa-361-5p	2.36E-02	hsa-miR-122-3p	8.04E-05
hsa-miR-377-3p	5.05E-04	hsa-miR-451a	2.51E-03	hsa-miR-320b	2.61E-02	hsa-miR-194-5p	3.18E-04
hsa-miR-99a-5p	1.17E-03	hsa-miR-146b-5p	3.18E-03	hsa-miR-26b-5p	2.62E-02	hsa-let-7b-5p	3.48E-04
hsa-miR-146b-5p	1.37E-03	hsa-miR-31-5p	4.34E-03	hsa-miR-320c	3.41E-02	hsa-miR-215	3.81E-04
hsa-miR-34a-5p	1.63E-03	hsa-miR-93-5p	9.20E-03	hsa-miR-24-3p	3.50E-02	hsa-let-7c	9.10E-04
hsa-miR-551b-3p	2.28E-03	hsa-miR-16-2-3p	1.04E-02	hsa-miR-376c	3.82E-02	hsa-miR-26a-5p	1.11E-03
hsa-miR-376c	2.57E-03	hsa-miR-10b-5p	1.07E-02	hsa-miR-342-3p	4.06E-02	hsa-miR-4492	1.48E-03
hsa-miR-100-5p	4.40E-03	hsa-miR-21-5p	1.10E-02	hsa-miR-29c-3p	4.72E-02	hsa-miR-4485	1.58E-03
hsa-miR-139-5p	1.03E-02	hsa-miR-425-5p	1.21E-02			hsa-miR-146a-5p	1.65E-03
hsa-miR-451a	1.15E-02	hsa-miR-16-5p	1.21E-02			hsa-miR-423-5p	1.65E-03
hsa-miR-23a-3p	1.30E-02	hsa-miR-34a-5p	1.29E-02			hsa-miR-181a-5p	1.76E-03
hsa-miR-144-5p	1.34E-02	hsa-miR-130a-3p	2.12E-02			hsa-miR-30b-5p	2.20E-03
hsa-miR-30a-3p	1.74E-02	hsa-miR-652-3p	2.16E-02			hsa-miR-644b-5p	2.44E-03
hsa-miR-22-5p	1.85E-02	hsa-miR-17-5p	2.38E-02			hsa-miR-1973	2.50E-03
hsa-miR-24-3p	2.05E-02	hsa-miR-106a-5p	2.41E-02			hsa-miR-214-3p	2.67E-03
hsa-miR-182-5p	2.22E-02	hsa-miR-127-3p	2.53E-02			hsa-miR-151b	4.33E-03
hsa-miR-342-3p	2.60E-02	hsa-miR-20a-5p	2.67E-02			hsa-miR-151a-5p	4.48E-03
hsa-miR-18a-5p	2.87E-02	hsa-miR-15b-5p	3.10E-02			hsa-miR-4668-5p	4.48E-03

hsa-miR-199b-5p	3.26E-02	hsa-miR-23a-3p	3.37E-02			hsa-miR-191-5p	4.56E-03
hsa-miR-148a-3p	3.35E-02	hsa-miR-25-3p	4.52E-02			hsa-miR-4301	5.39E-03
hsa-miR-199a-5p	3.55E-02					hsa-miR-3613-3p	5.93E-03
hsa-miR-22-3p	3.63E-02					hsa-miR-4505	7.15E-03
hsa-miR-486-5p	3.65E-02					hsa-miR-513a-5p	8.79E-03
hsa-miR-4306	3.88E-02					hsa-let-7a-5p	8.79E-03
hsa-miR-185-5p	4.75E-02					hsa-miR-4284	9.74E-03
hsa-miR-363-3p	5.00E-02					hsa-miR-5001-5p	1.13E-02
						hsa-miR-663a	1.15E-02
						hsa-miR-3656	1.52E-02
Downregulated	$p < 0.001$					hsa-miR-4508	1.77E-02
Upregulated	$p < 0.01$					hsa-let-7d-5p	1.81E-02
Too little signal	$p < 0.05$					hsa-miR-4443	2.22E-02
						hsa-miR-335-5p	2.24E-02
						hsa-miR-363-5p	2.27E-02
						hsa-miR-4656	2.33E-02
						hsa-miR-4417	2.33E-02
						hsa-miR-195-5p	2.53E-02
						hsa-miR-3621	2.79E-02
						hsa-miR-3687	2.96E-02
						hsa-miR-1273f	3.06E-02
						hsa-miR-3182	3.08E-02
						hsa-miR-1587	3.10E-02
						hsa-miR-4787-5p	3.30E-02
						hsa-miR-30a-5p	4.40E-02
						hsa-miR-1273c	4.50E-02
						hsa-miR-4516	4.73E-02
						hsa-miR-4507	4.77E-02

						hsa-miR-4280	4.82E-02
						hsa-miR-130a-3p	4.88E-02
						hsa-miR-195-3p	1.63E-04
						hsa-miR-1248	3.47E-04
						hsa-miR-3183	7.63E-03
						hsa-miR-572	2.24E-02

Legends for Supplementary Figures:

Figure S1: *Regulation network of DEmiRNAs in CAD*: Visualization of the mRNA targets of DEmiRNAs in EAT of patients with CAD compared to non-CAD. Network was generated using ClueGO v2.5.8 and CluePedia v1.5.8 in Cytoscape v3.9.0 with the miRTarBase set of verified miRNA targets.

Figure S2: *GO enrichment*: GO enrichment analysis performed using ClueGO v2.5.8 and CluePedia v1.5.8 in Cytoscape v3.9.0, shows the biological processes and cellular components targeted by the DEmiRNAs in the EAT of CAD patients. The graph shows the percent of gene targets represented in the GO pathway.

Figure S3: *Gene Ontology (GO) category clustering of EAT DEmiRNAs in CAD.* The GO categories displayed were produced using the targets of the miRNAs that were differentially expressed in the EAT of patients with CAD (from the network in **Figure S1**) in Cytoscape v3.9.0 using the tools ClueGO v2.5.8 and CluePedia v1.5.8. The coloration of the nodes corresponds to the same categories depicted in **Figure 5A** and **Figure S2**.

Figure S4: *KEGG pathway enrichment*: The figure produced using ClueGO v2.5.8 and CluePedia v1.5.8 in Cytoscape v3.9.0 shows the percent of gene targets of DEmiRNAs falling into each of the represented KEGG pathway.

Figure S5: *Clustering of Enriched KEGG Pathways of the EAT DEmiRNAS in CAD*. The KEGG pathways displayed were produced using the targets of the miRNAs that were differentially expressed in the EAT of patients with coronary artery disease (from the network in **Figure S1**) in Cytoscape v3.9.0 using the tools ClueGO v2.5.8 and CluePedia v1.5.8. The coloration of the nodes corresponds to the same categories depicted in **Figure 5B** and **Figure S4**.

Figure S6: *Validation of DEmRNA and DEmiRNA in EAT Between Patients with and without CAD***:** A – Validation of the differential expression of DUSP4 and FOXO3 in epicardial adipose tissue; B – Validation of the differential expression of select microRNAs in epicardial adipose tissue. Significance was determined using unpaired T-Test to compare the ΔC_t values obtained from qPCR. The graphs display the fold change in tissue from patients with coronary artery disease in comparison to patients without coronary artery disease. hsa-miR-146b-5p and hsa-miR-26b-5p were highly downregulated in the epicardial adipose tissue of patients with coronary artery disease, but this downregulation was not statistically significant ($p \leq 0.05$).

Figure S1.

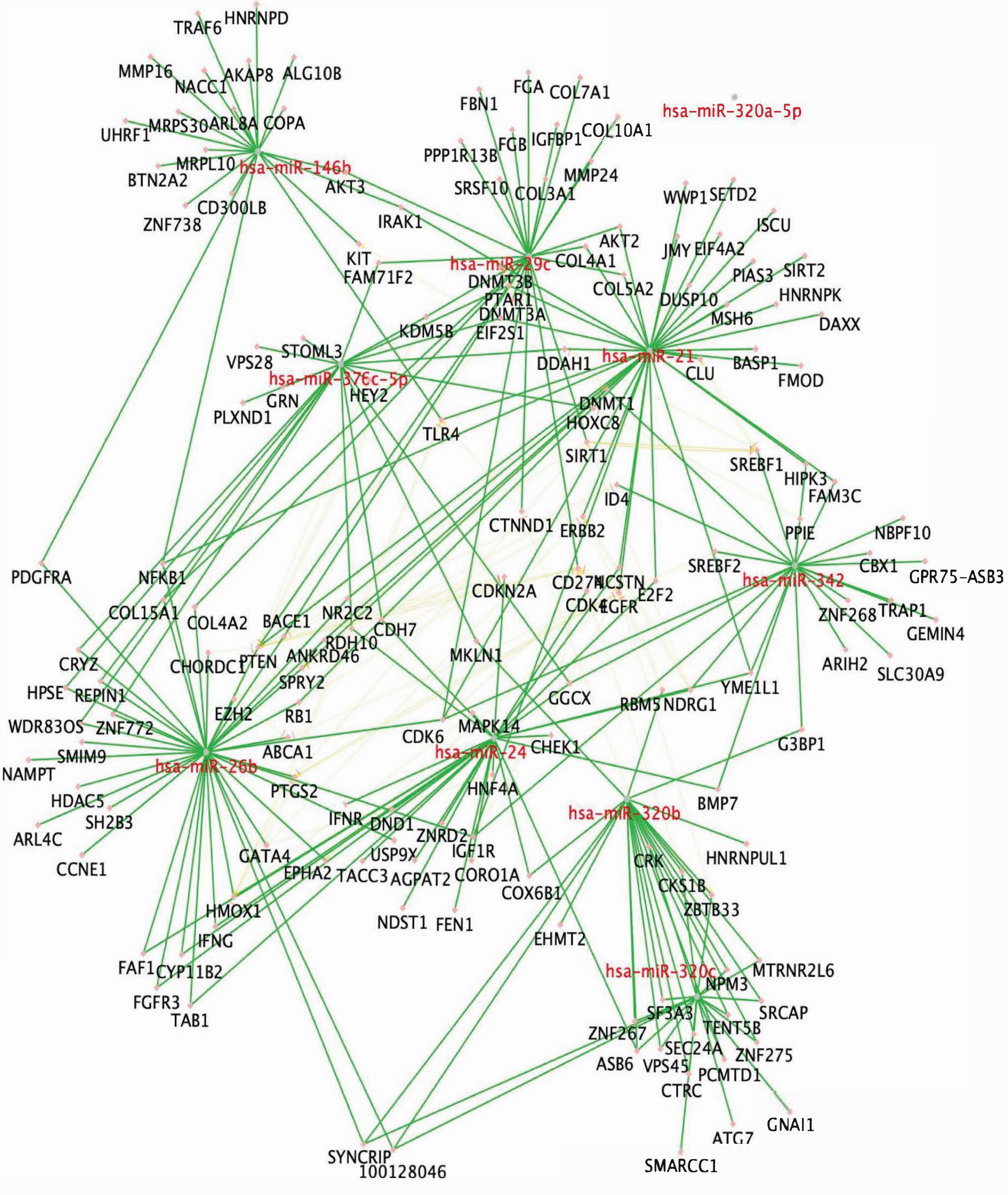
TRAF6
HNRNPD
MMP16
NACC1
AKAP8
ALG10B
UHRF1
MRPS30
ARL8A
COPA
MRPL10
hsa-miR-146b
BTN2A2
AKT3
CD300LB
ZNF738
IRAK1
FBN1
FGA
COL7A1
FGB
IGFBP1
COL10A1
PPP1R13B
MMP24
SRSF10
COL3A1
hsa-miR-320a-5p
WWP1
SETD2
ISCU
AKT2
JMY
EIF4A2
KIT
hsa-miR-29c
COL4A1
PIAS3
SIRT2
FAM71F2
DNMT3B
COL5A2
DUSP10
HNRNPK
PTAR1
DNMT3A
MSH6
DAXX
KDM5B
EIF2S1
STOML3
hsa-miR-21
VPS28
DDAH1
CLU
BASP1
FMOD
hsa-miR-376c-5p
HEY2
GRN
DNMT1
PLXND1
HOXC8
TLR4
SIRT1
SREBF1
HIPK3
FAM3C
ID4
CTNND1
ERBB2
PPIE
NBPF10
CBX1
GPR75-ASB3
PDGFRA
NFKB1
SREBF2
hsa-miR-342
CD274
NCSTN
CDKN2A
E2F2
COL15A1
CDK4
EGFR
TRAP1
ZNF268
GEMIN4
COL4A2
BACE1
NR2C2
CDH7
RDH10
ARIH2
SLC30A9
CRYZ
PTEN
ANKRD46
MKLN1
CHORDC1
SPRY2
HPSE
REPIN1
YME1L1
GGCX
RBM5
NDRG1
EZH2
RB1
WDR83OS
ZNF772
CDK6
MAPK14
CHEK1
G3BP1
SMIM9
ABCA1
hsa-miR-24
NAMPT
hsa-miR-26b
HNF4A
HDAC5
PTGS2
BMP7
SH2B3
IFNR
DND1
hsa-miR-320b
ARL4C
ZNRD2
IGF1R
HNRNPUL1
CCNE1
GATA4
USP9X
CRK
EPHA2
TACC3
AGPAT2
CORO1A
CKS1B
COX6B1
ZBTB33
HMOX1
NDST1
FEN1
IFNG
EHMT2
FAF1
CYP11B2
hsa-miR-320c
MTRNR2L6
NPM3
FGFR3
SF3A3
SRCAP
TAB1
ZNF267
TENT5B
SEC24A
ZNF275
ASB6
VPS45
PCMTD1
CTRC
GNAI1
ATG7
SYNCRIP
100128046
SMARCC1

Figure S2.

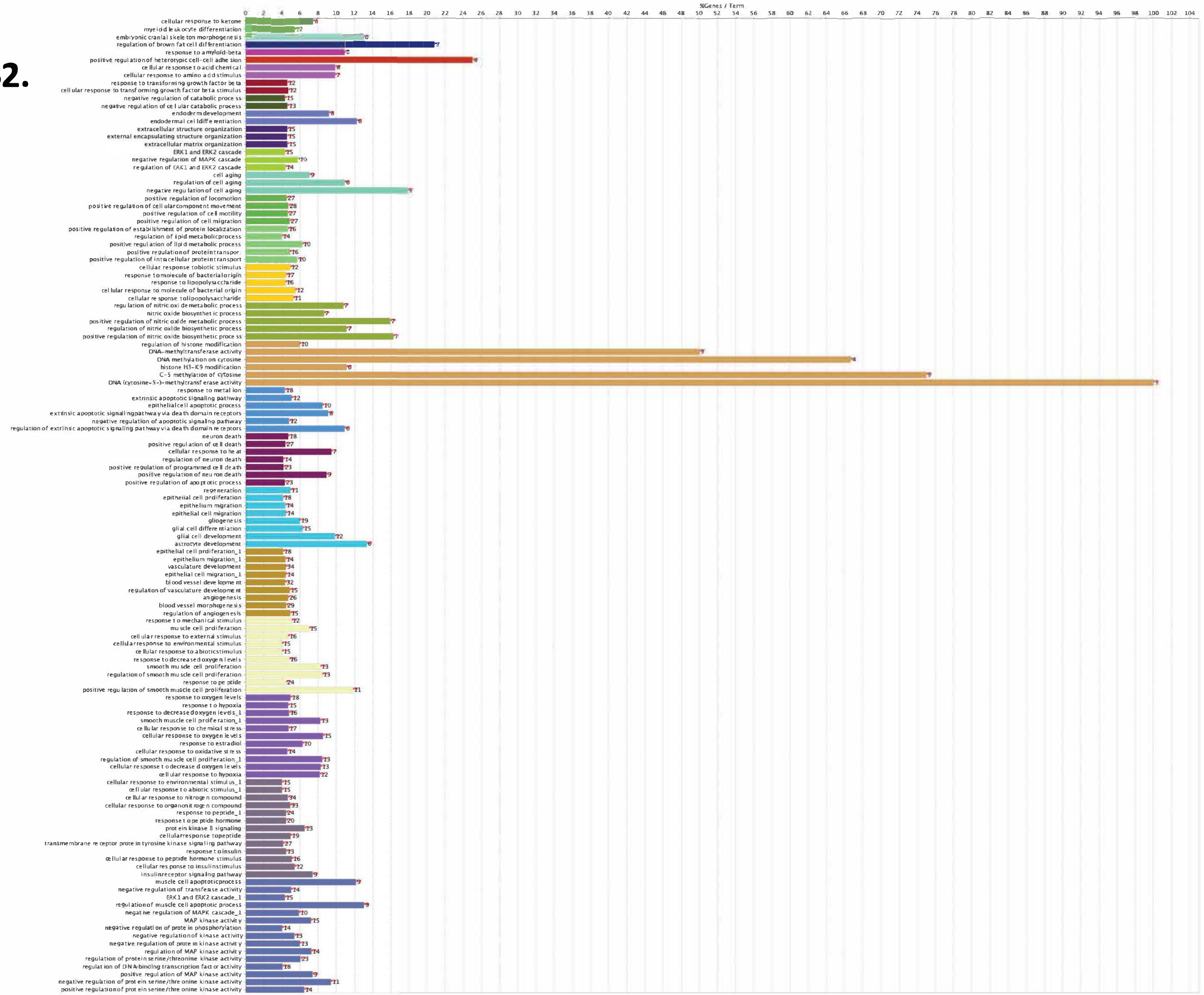

%Genes / Term
0
2
4
6
8
10
12
14
16
18
20
22
24
26
28
30
32
34
36
38
40
42
44
46
48
50
52
54
56
58
60
62
64
66
68
70
72
74
76
78
80
82
84
86
88
90
92
94
96
98
100
102
104
cellular response to ketone
myeloid leukocyte differentiation
embryonic cranial skeleton morphogenesis
regulation of brown fat cell differentiation
response to amyloid-beta
positive regulation of heterotypic cell-cell adhesion
cellular response to acid chemical
cellular response to amino acid stimulus
response to transforming growth factor beta
cellular response to transforming growth factor beta stimulus
negative regulation of catabolic process
negative regulation of cellular catabolic process
endoderm development
endodermal cell differentiation
extracellular structure organization
external encapsulating structure organization
extracellular matrix organization
ERK1 and ERK2 cascade
negative regulation of MAPK cascade
regulation of ERK1 and ERK2 cascade
cell aging
regulation of cell aging
negative regulation of cell aging
positive regulation of locomotion
positive regulation of cellular component movement
positive regulation of cell motility
positive regulation of cell migration
positive regulation of establishment of protein localization
regulation of lipid metabolic process
positive regulation of lipid metabolic process
positive regulation of protein transport
positive regulation of intracellular protein transport
cellular response to biotic stimulus
response to molecule of bacterial origin
response to lipopolysaccharide
cellular response to molecule of bacterial origin
cellular response to lipopolysaccharide
regulation of nitric oxide metabolic process
nitric oxide biosynthetic process
positive regulation of nitric oxide metabolic process
regulation of nitric oxide biosynthetic process
positive regulation of nitric oxide biosynthetic process
regulation of histone modification
DNA-methyltransferase activity
DNA methylation on cytosine
histone H3-K9 modification
C-5 methylation of cytosine
DNA (cytosine-5-)-methyltransferase activity
response to metal ion
extrinsic apoptotic signaling pathway
epithelial cell apoptotic process
extrinsic apoptotic signaling pathway via death domain receptors
negative regulation of apoptotic signaling pathway
regulation of extrinsic apoptotic signaling pathway via death domain receptors
neuron death
positive regulation of cell death
cellular response to heat
regulation of neuron death
positive regulation of programmed cell death
positive regulation of neuron death
positive regulation of apoptotic process
regeneration
epithelial cell proliferation
epithelium migration
epithelial cell migration
gliogenesis
glial cell differentiation
glial cell development
astrocyte development
epithelial cell proliferation_1
epithelium migration_1
vasculature development
epithelial cell migration_1
blood vessel development
regulation of vasculature development
angiogenesis
blood vessel morphogenesis
regulation of angiogenesis
response to mechanical stimulus
muscle cell proliferation
cellular response to external stimulus
cellular response to environmental stimulus
cellular response to abiotic stimulus
response to decreased oxygen levels
smooth muscle cell proliferation
regulation of smooth muscle cell proliferation
response to peptide
positive regulation of smooth muscle cell proliferation
response to oxygen levels
response to hypoxia
response to decreased oxygen levels_1
smooth muscle cell proliferation_1
cellular response to chemical stress
cellular response to oxygen levels
response to estradiol
cellular response to oxidative stress
regulation of smooth muscle cell proliferation_1
cellular response to decreased oxygen levels
cellular response to hypoxia
cellular response to environmental stimulus_1
cellular response to abiotic stimulus_1
cellular response to nitrogen compound
cellular response to organonitrogen compound
response to peptide_1
response to peptide hormone
protein kinase B signaling
cellular response to peptide
transmembrane receptor protein tyrosine kinase signaling pathway
response to insulin
cellular response to peptide hormone stimulus
cellular response to insulin stimulus
insulin receptor signaling pathway
muscle cell apoptotic process
negative regulation of transferase activity
ERK1 and ERK2 cascade_1
regulation of muscle cell apoptotic process
negative regulation of MAPK cascade_1
MAP kinase activity
negative regulation of protein phosphorylation
negative regulation of kinase activity
negative regulation of protein kinase activity
regulation of MAP kinase activity
regulation of protein serine/threonine kinase activity
regulation of DNA-binding transcription factor activity
positive regulation of MAP kinase activity
negative regulation of protein serine/threonine kinase activity
positive regulation of protein serine/threonine kinase activity

Figure S3.

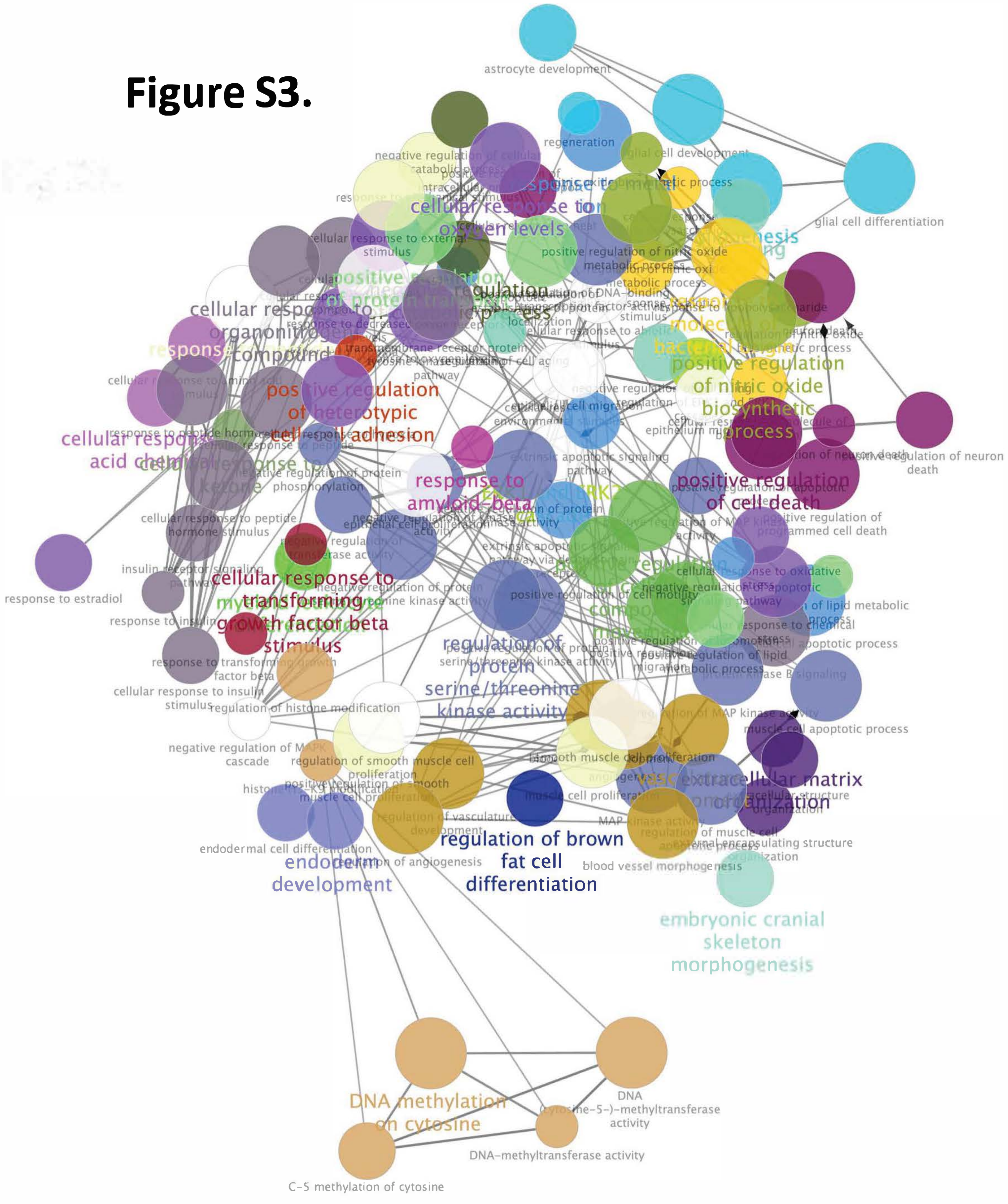

astrocyte development
regeneration
glial cell development
glial cell differentiation
cellular response to
oxygen levels
positive regulation of nitric oxide
metabolic process
cellular response to external
stimulus
cellular response to
organonitrogen
compound
positive regulation
of heterotypic
cell-cell adhesion
positive regulation
of nitric oxide
biosynthetic
process
cellular response to amino acid
stimulus
response to estradiol
response to insulin
cellular response to peptide
hormone stimulus
insulin receptor signaling
pathway
response to
amyloid-beta
positive regulation
of cell death
positive regulation of neuron
death
positive regulation of
programmed cell death
cellular response to
transforming
growth factor beta
stimulus
response to transforming growth
factor beta
cellular response to insulin
stimulus
regulation of histone modification
negative regulation of MAPK
cascade
regulation of smooth muscle cell
proliferation
regulation of
protein
serine/threonine
kinase activity
muscle cell apoptotic process
extracellular matrix
organization
external encapsulating structure
organization
endodermal cell differentiation
endoderm
development
regulation of brown
fat cell
differentiation
blood vessel morphogenesis
embryonic cranial
skeleton
morphogenesis
DNA methylation
on cytosine
DNA
(cytosine-5-)-methyltransferase
activity
DNA-methyltransferase activity
C-5 methylation of cytosine

Figure S4.

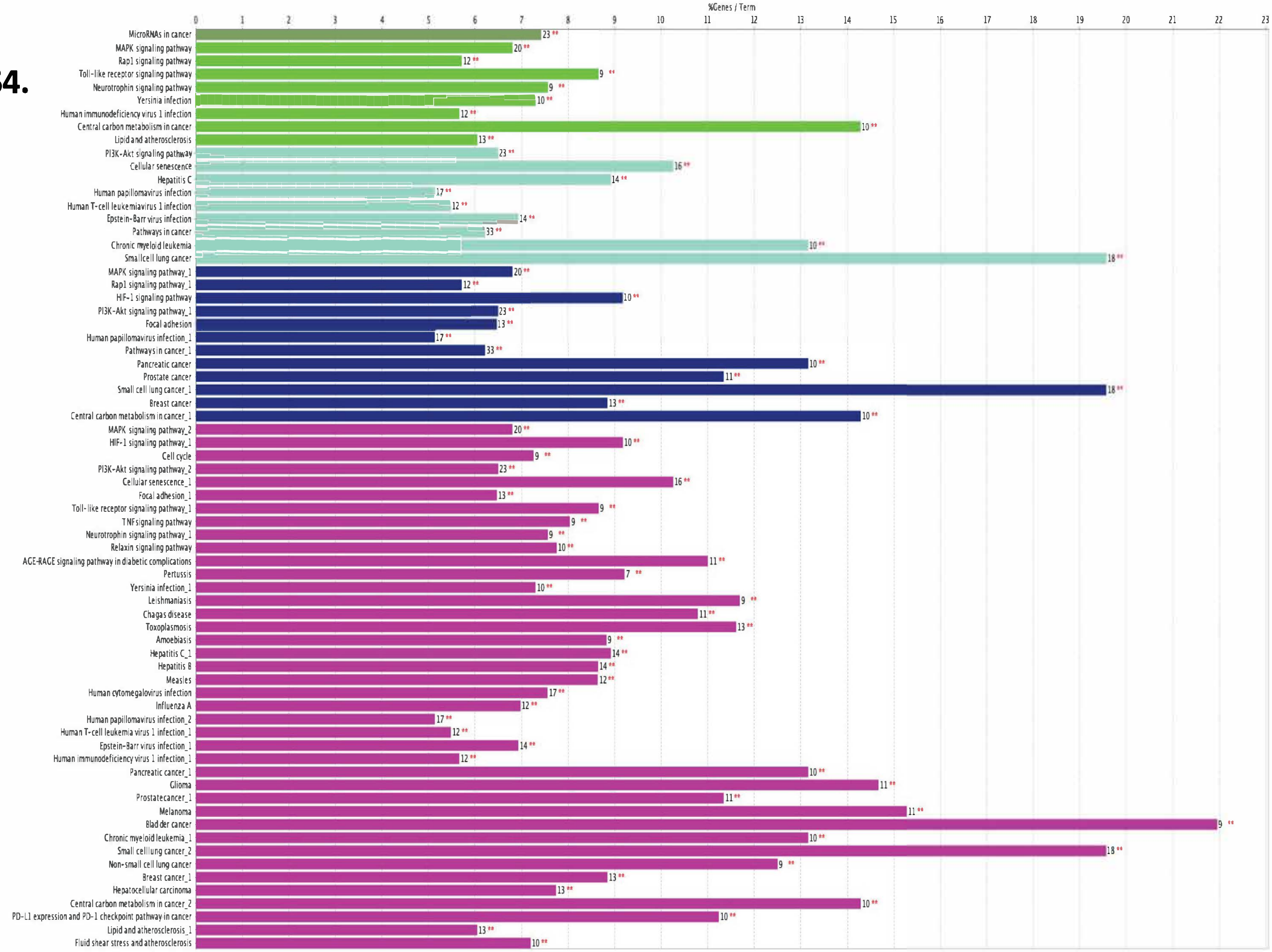
%Genes / Term
0 1 2 3 4 5 6 7 8 9 10 11 12 13 14 15 16 17 18 19 20 21 22 23
MicroRNAs in cancer 23 **
MAPK signaling pathway 20 **
Rap1 signaling pathway 12 **
Toll-like receptor signaling pathway 9 **
Neurotrophin signaling pathway 9 **
Yersinia infection 10 **
Human immunodeficiency virus 1 infection 12 **
Central carbon metabolism in cancer 10 **
Lipid and atherosclerosis 13 **
PI3K-Akt signaling pathway 23 **
Cellular senescence 16 **
Hepatitis C 14 **
Human papillomavirus infection 17 **
Human T-cell leukemia virus 1 infection 12 **
Epstein-Barr virus infection 14 **
Pathways in cancer 33 **
Chronic myeloid leukemia 10 **
Small cell lung cancer 18 **
MAPK signaling pathway_1 20 **
Rap1 signaling pathway_1 12 **
HIF-1 signaling pathway 10 **
PI3K-Akt signaling pathway_1 23 **
Focal adhesion 13 **
Human papillomavirus infection_1 17 **
Pathways in cancer_1 33 **
Pancreatic cancer 10 **
Prostate cancer 11 **
Small cell lung cancer_1 18 **
Breast cancer 13 **
Central carbon metabolism in cancer_1 10 **
MAPK signaling pathway_2 20 **
HIF-1 signaling pathway_1 10 **
Cell cycle 9 **
PI3K-Akt signaling pathway_2 23 **
Cellular senescence_1 16 **
Focal adhesion_1 13 **
Toll-like receptor signaling pathway_1 9 **
TNF signaling pathway 9 **
Neurotrophin signaling pathway_1 9 **
Relaxin signaling pathway 10 **
AGE-RAGE signaling pathway in diabetic complications 11 **
Pertussis 7 **
Yersinia infection_1 10 **
Leishmaniasis 9 **
Chagas disease 11 **
Toxoplasmosis 13 **
Amoebiasis 9 **
Hepatitis C_1 14 **
Hepatitis B 14 **
Measles 12 **
Human cytomegalovirus infection 17 **
Influenza A 12 **
Human papillomavirus infection_2 17 **
Human T-cell leukemia virus 1 infection_1 12 **
Epstein-Barr virus infection_1 14 **
Human immunodeficiency virus 1 infection_1 12 **
Pancreatic cancer_1 10 **
Glioma 11 **
Prostate cancer_1 11 **
Melanoma 11 **
Bladder cancer 9 **
Chronic myeloid leukemia_1 10 **
Small cell lung cancer_2 18 **
Non-small cell lung cancer 9 **
Breast cancer_1 13 **
Hepatocellular carcinoma 13 **
Central carbon metabolism in cancer_2 10 **
PD-L1 expression and PD-1 checkpoint pathway in cancer 10 **
Lipid and atherosclerosis_1 13 **
Fluid shear stress and atherosclerosis 10 **

Figure S5.

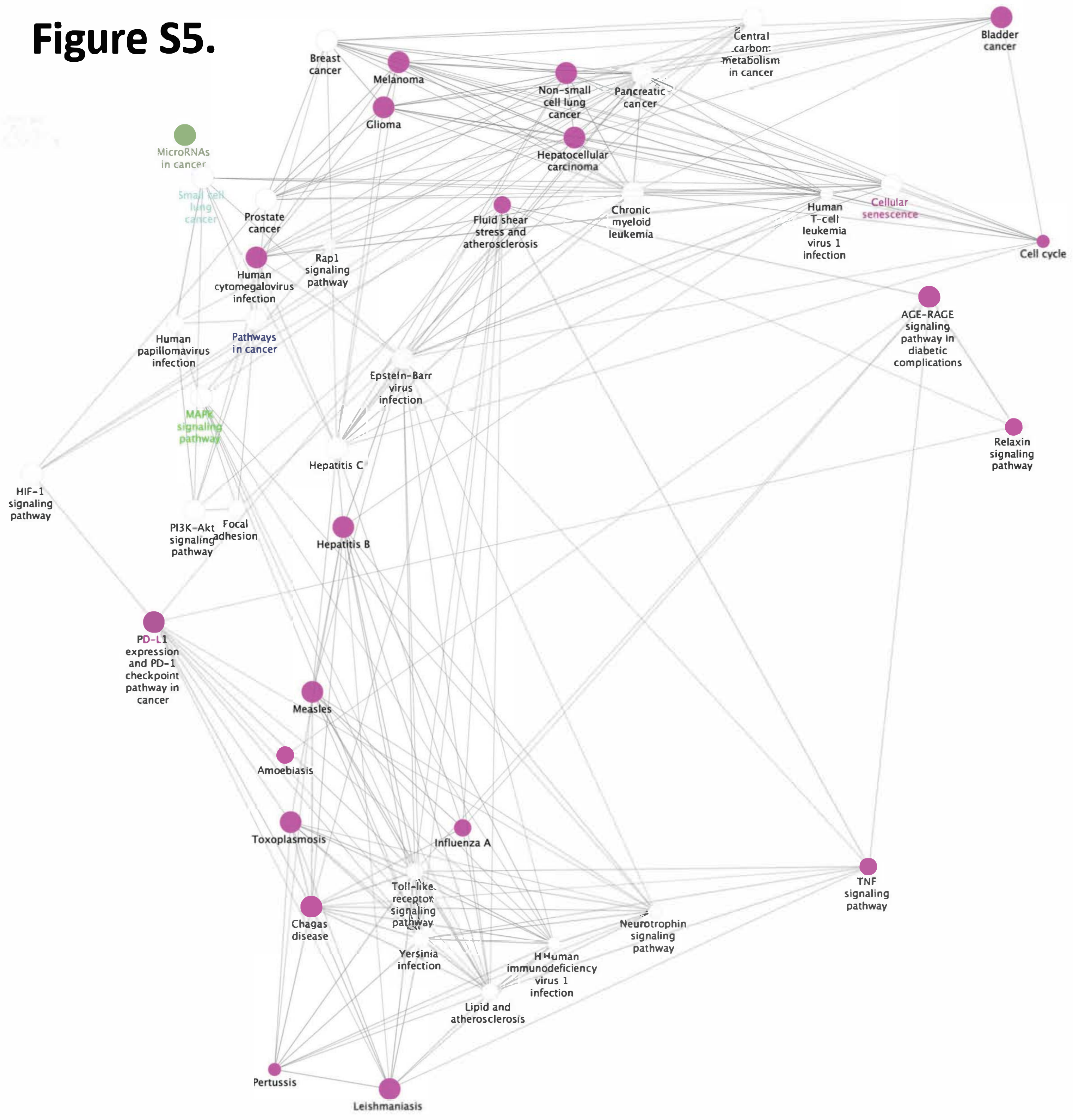

Breast cancer
Melanoma
Central carbon metabolism in cancer
Bladder cancer
Non-small cell lung cancer
Pancreatic cancer
Glioma
MicroRNAs in cancer
Hepatocellular carcinoma
Small cell lung cancer
Prostate cancer
Fluid shear stress and atherosclerosis
Chronic myeloid leukemia
Human T-cell leukemia virus 1 infection
Cellular senescence
Cell cycle
Human cytomegalovirus infection
Rap1 signaling pathway
AGE-RAGE signaling pathway in diabetic complications
Human papillomavirus infection
Pathways in cancer
Epstein-Barr virus infection
MAPK signaling pathway
Relaxin signaling pathway
Hepatitis C
HIF-1 signaling pathway
PI3K-Akt signaling pathway
Focal adhesion
Hepatitis B
PD-L1 expression and PD-1 checkpoint pathway in cancer
Measles
Amoebiasis
Toxoplasmosis
Influenza A
TNF signaling pathway
Toll-like receptor signaling pathway
Chagas disease
Neurotrophin signaling pathway
Yersinia infection
Human immunodeficiency virus 1 infection
Lipid and atherosclerosis
Pertussis
Leishmaniasis

Figure S6.

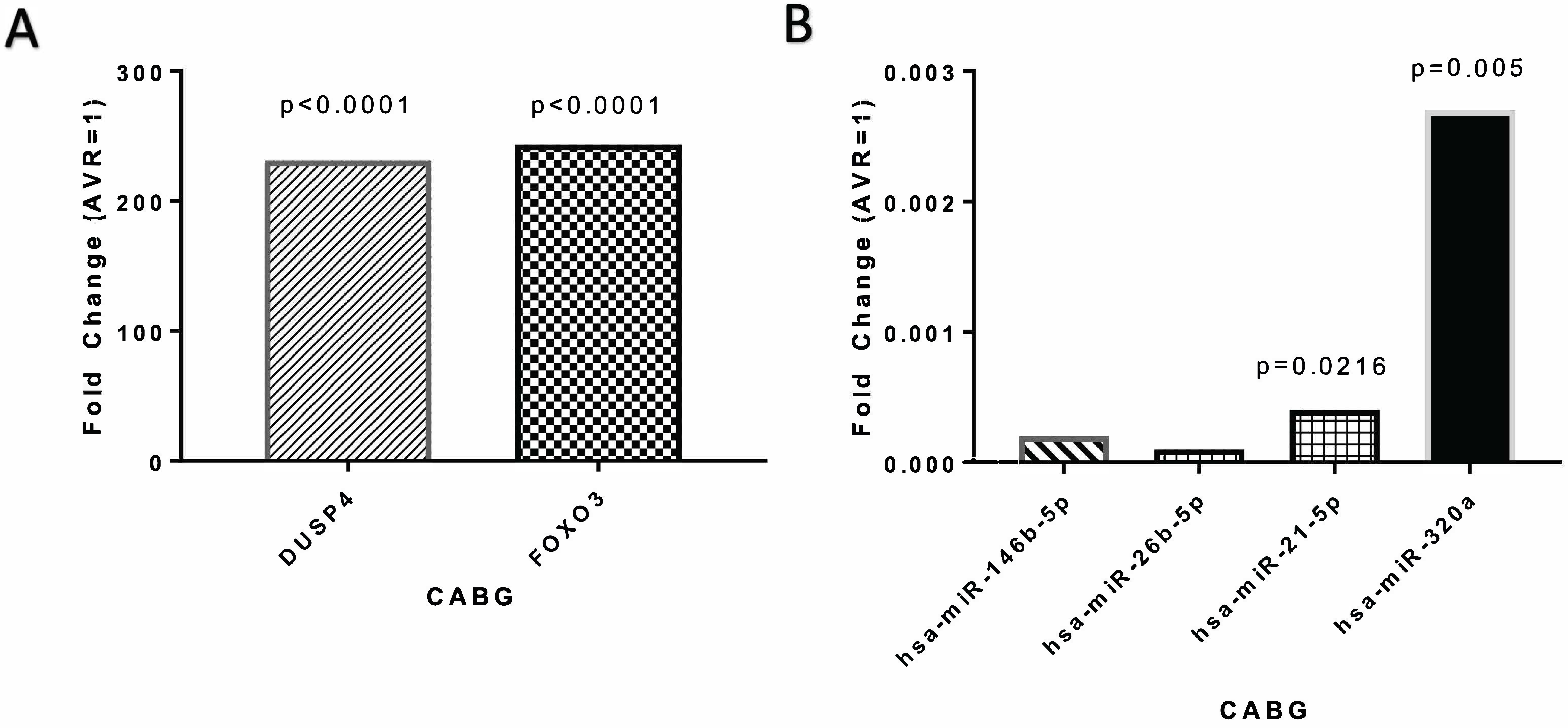
A
300
200
100
0
Fold Change (AVR=1)
p<0.0001
p<0.0001
DUSP4
FOXO3
CABG
B
0.003
0.002
0.001
0.000
Fold Change (AVR=1)
p=0.005
p=0.0216
hsa-miR-146b-5p
hsa-miR-26b-5p
hsa-miR-21-5p
hsa-miR-320a
CABG
